# Supplementary material for: Decoding the epigenetics and chromatin loop dynamics of androgen receptor-mediated transcription
Source: Nat Commun. 2024 Nov 3;15:9494. doi: 10.1038/s41467-024-53758-5 (PMC11532539; doi:10.1038/s41467-024-53758-5)
Supplement: Supplementary file 1 — Supplementary Information [file 41467_2024_53758_MOESM1_ESM.pdf]

Supp Fig 1

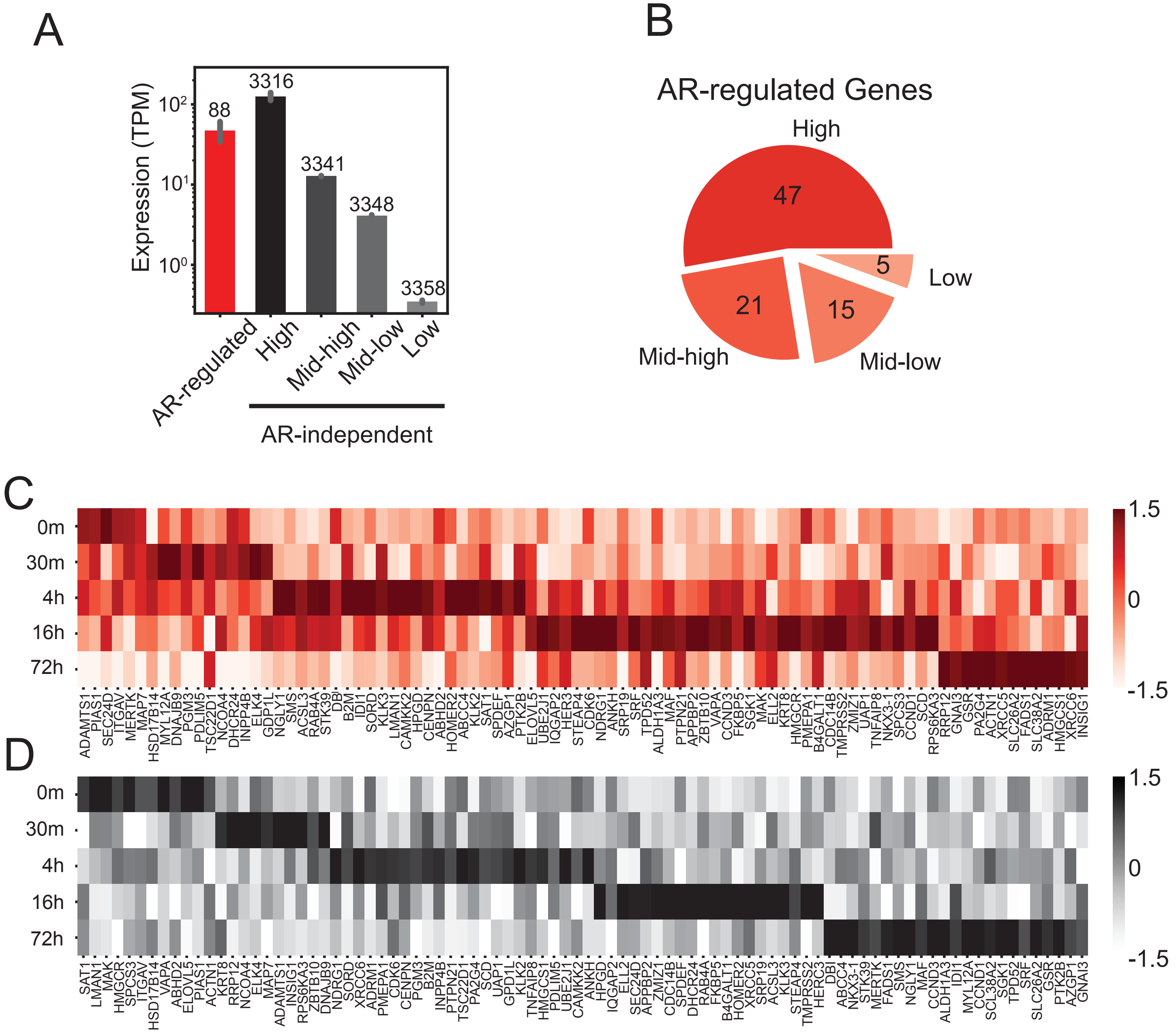

Supplementary Figure 1: We split the transcriptome into four equal-size groups according to their average expresion. (A) The average expression of AR-upregulated genes (red) and AR-independent gene quartiles (greys). **(B)** Pie chart illustrating the distribution of AR-upregulated genes among assigned quartiles. **(C, D)** Heatmaps (RNA-seq, and Start-seq; respectively) representing z-score expression profile of AR-upregulated genes in time.

Supp Fig 2

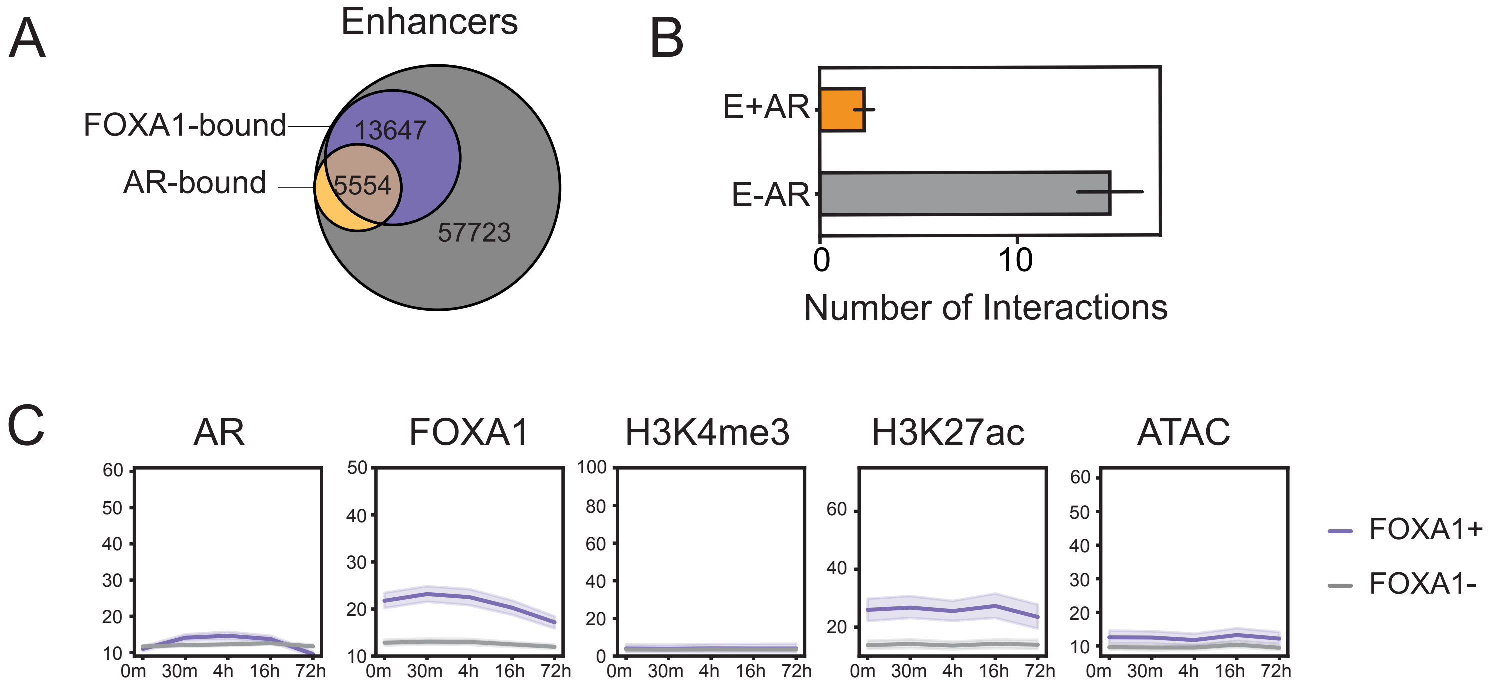

Supplementary Figure 2: FOXA1 signal is not affected when AR is absent. (A) Venn diagram representing AR-bound, FOXA1-bound enhancers. **(B)** Number of E+AR and E-AR loops of AR-regulated genes. **(C)** Epigenome changes (see Fig 2) of AR-free enhancers that are either FOXA1-bound (purple) or FOXA1-free (grey).

Supp Fig 3

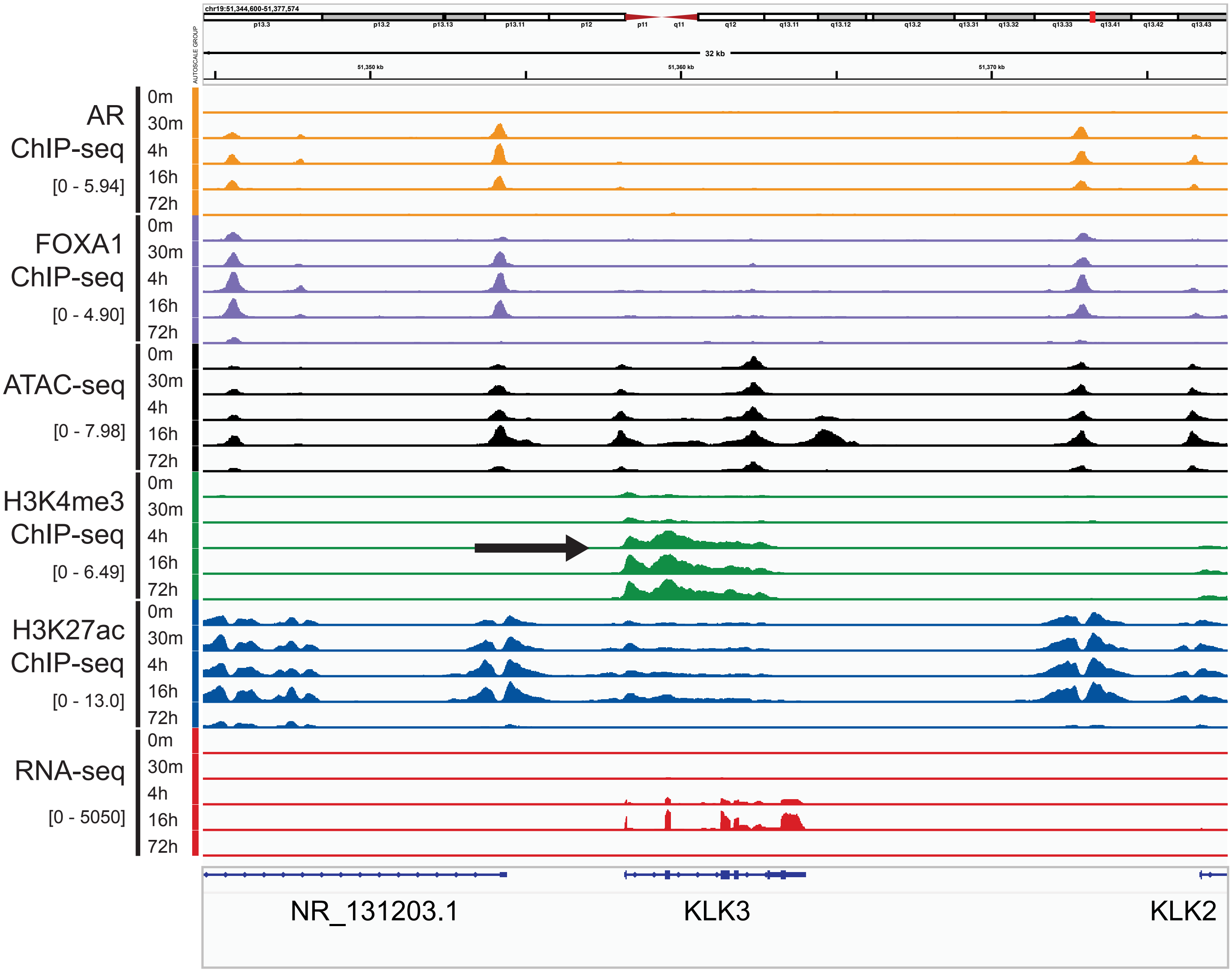

Supplementary Figure 3: **IGV track for KLK3 (~15kb) gene locus.** AR (yellow), FOXA1 (purple), ATAC-seq (black), H3K4me3 (green), H3K27ac (blue), RNA-seq (red) tracks. The arrow represents the H3K4me3 signal at 4h on the KLK3 promoter.

Supp Fig 4

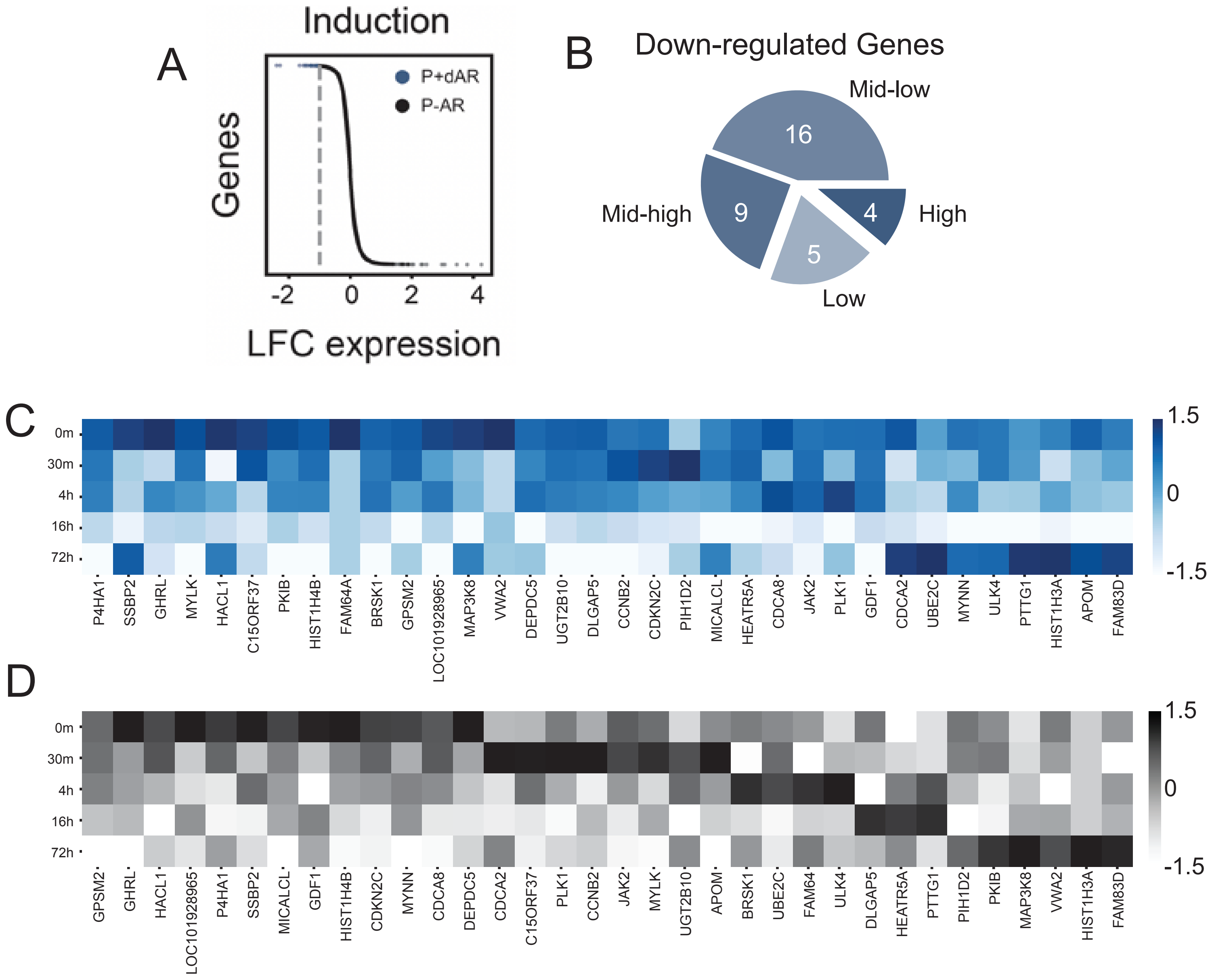

Supplementary Figure 4: Identification of down regulated genes: (A) The downregulated genes are selected according to 16h vs 0m Log2FoldChange (-1 >). **(B)** The number of genes in each quartile. **(C, D)** Heatmaps (RNA-seq, and Start-seq; respectively) representing z-score expression profile of AR-downregulated genes in time.

Supp Fig 5

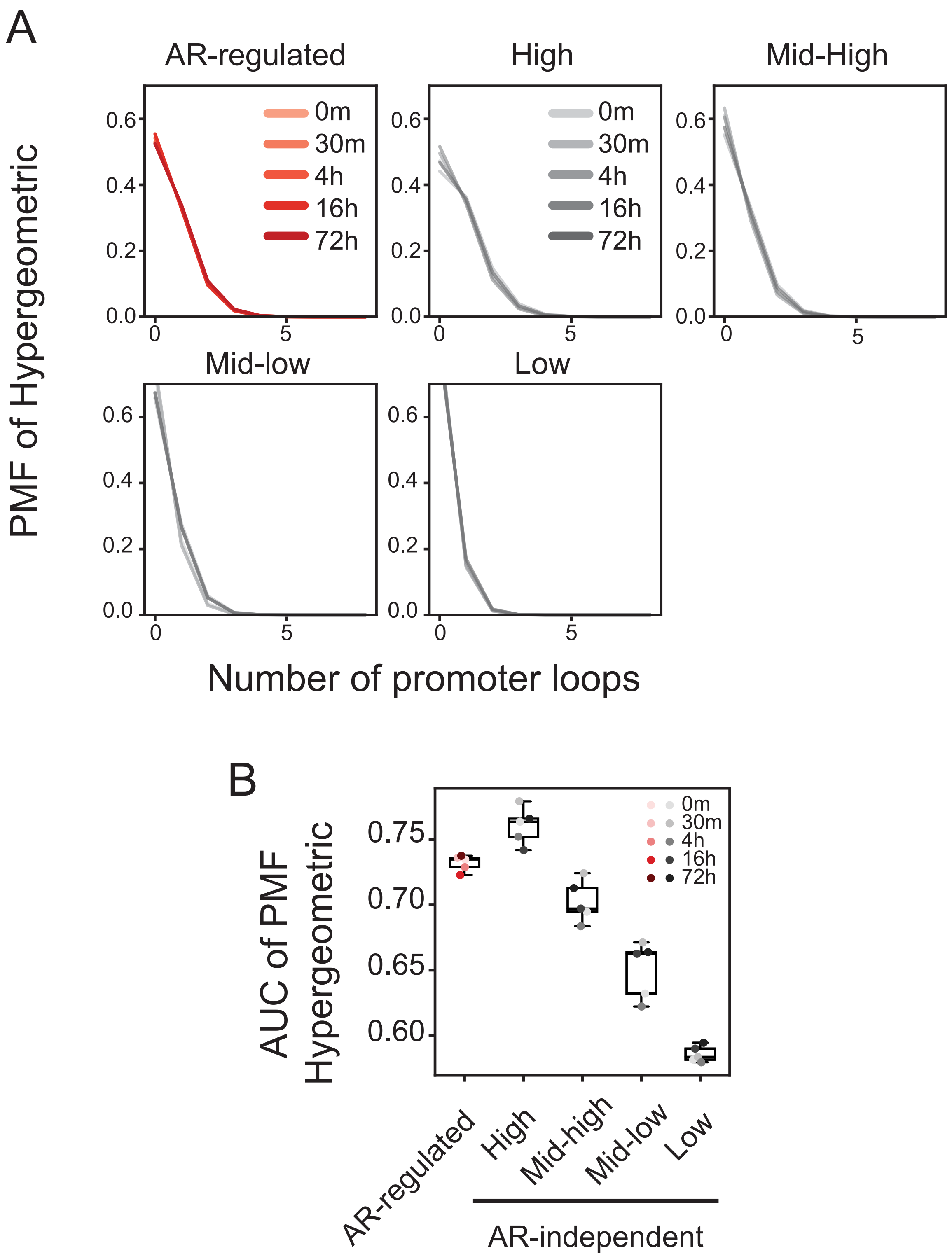

Supplementary Figure 5: Androgen treatmen does not change the number of loop, the loop number changes with gene expression. (A) Probability mass function (PMF) of hypergeometric distribution of the number of loops on gene promoters for each time point. Each pane represents one gene set (AR-regulated or expression quartiles). **(B)** The area-under-curve (AUC) of the PMF of hypergeometric (see A).

Supp Fig 6

A

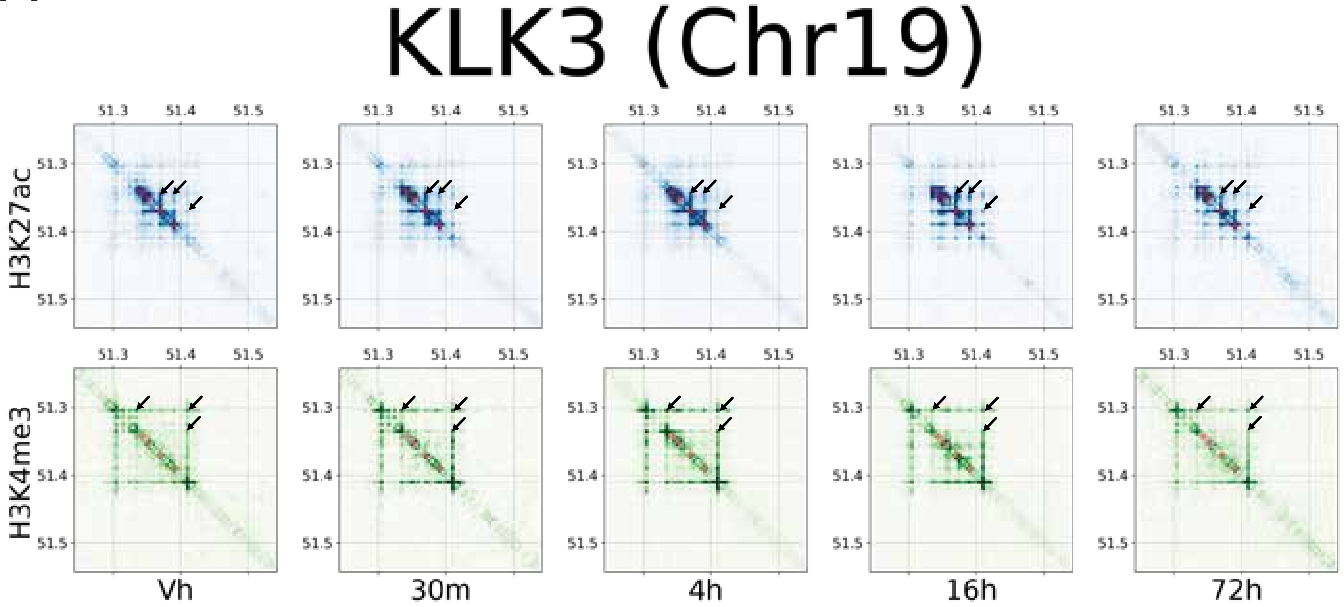

B

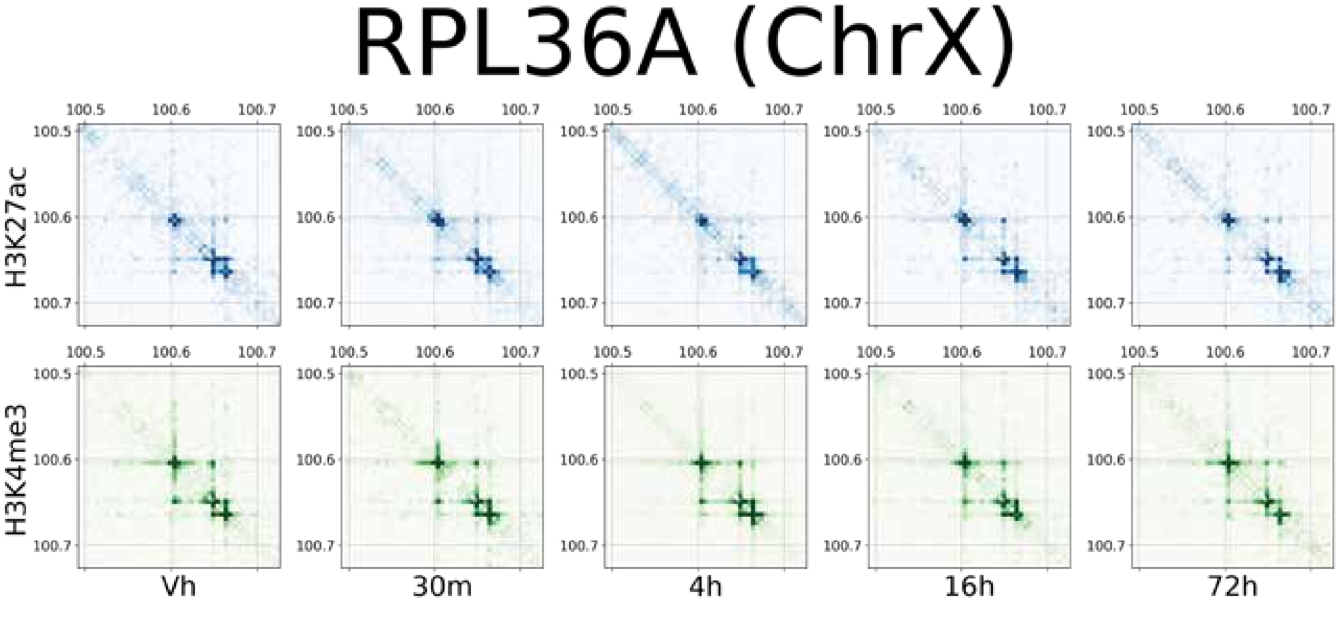

Supplementary Figure 6: Contact maps of H3K27ac (top) and H3K4me3 (bottom) centering the gene promoters (white) and their putative AR-bound enhancers. +/- 150kb with 5kb resolution **(A)** KLK3 locus, an androgen responsive gene. The arrows represent increasing contact frequency of AR-bound enhancer loops. **(B)** RPL36A locus, a highly expressed gene that has no AR-bound enhancers.

Supp Fig 7

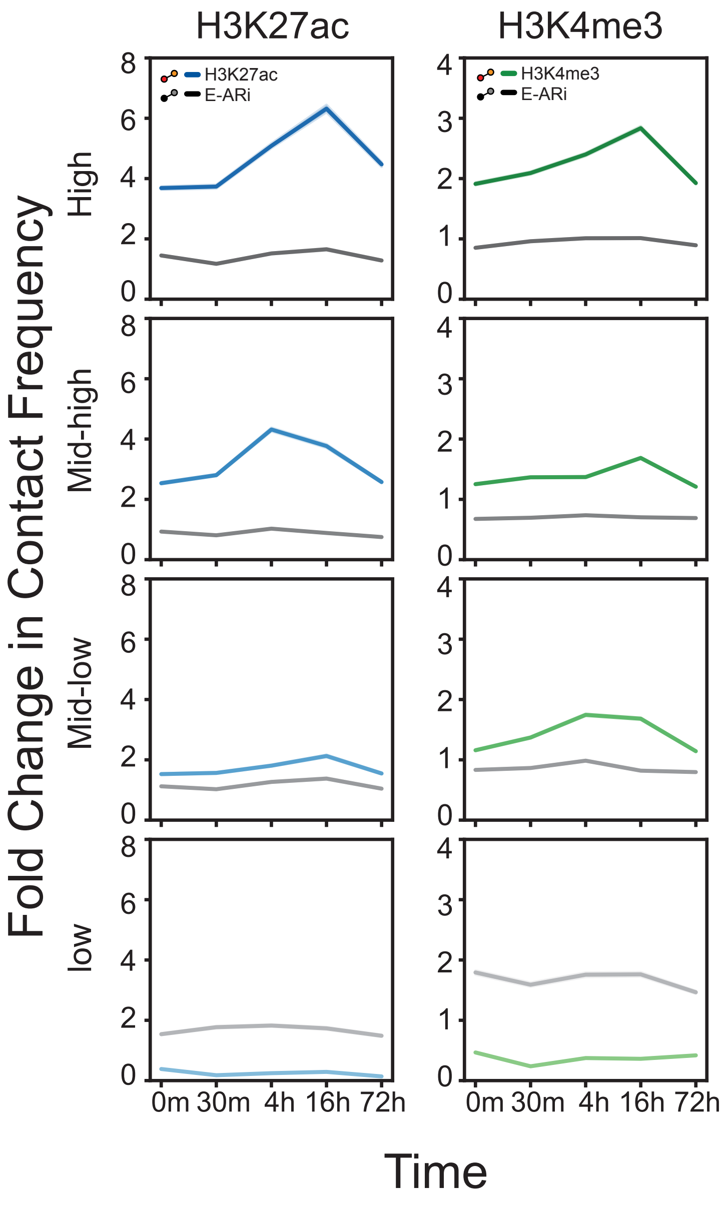

Supplementary Figure 7: Fold change in contact frequency of AR-bound enhancers looping to genes at differnt expression quartiles (rows) for H3K27ac (E+AR; blue) and H3K4me3 (E+AR; green) HiChIP. These CREs were compared to AR-free enhancers of highly expressed genes (E-ARi; black) in both HiChIP datasets.

Supp Fig 8

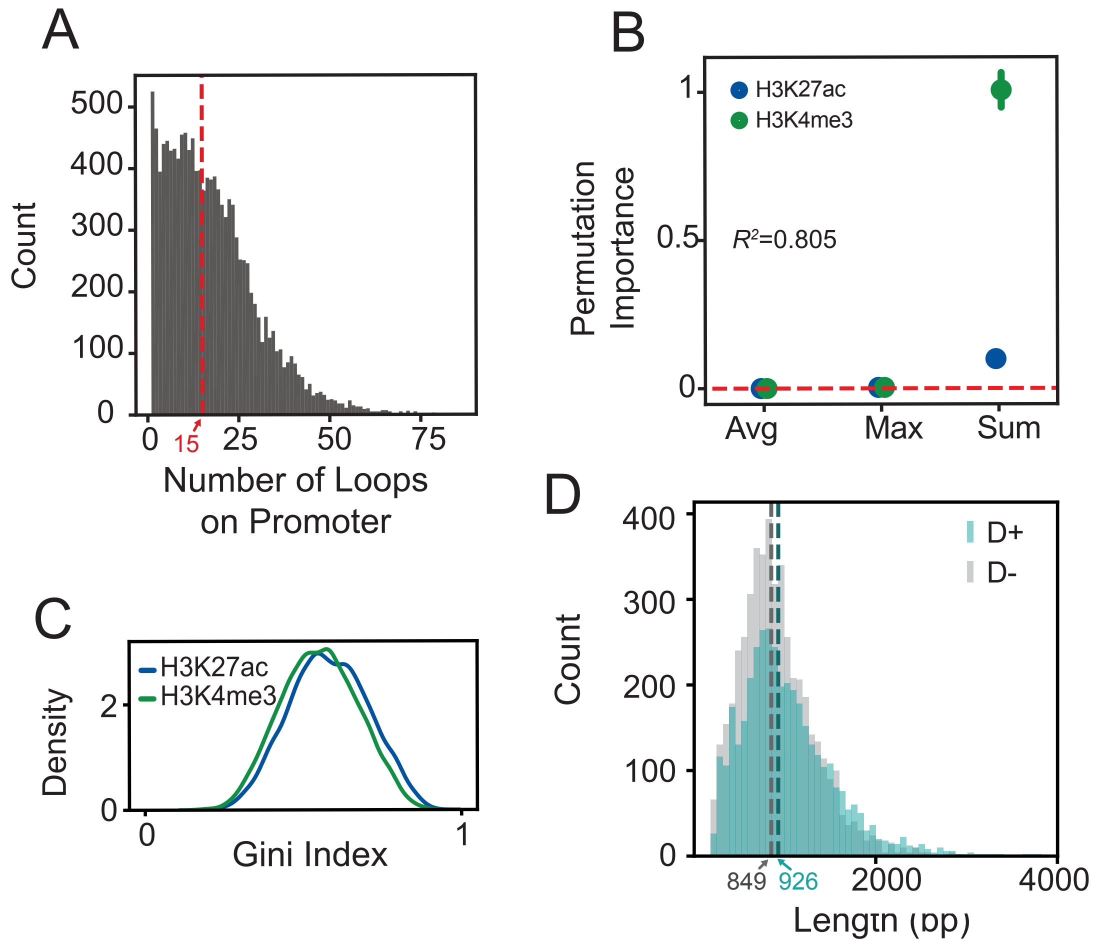

Supplementary Figure 8: **Dominance model. (A)** Number of loops on gene promoters. **(B)** Random forest regressor feature permutation importance. **(C)** Density plot representing Gini-index for AR-regulated (P+AR) and AR-independent (P-AR) gene promoters. **(D)** Length distribution of accessible sites (ATAC-seq) within dominant (D+) and non-dominant (D-) AR-bound elements.

Supp Fig 9

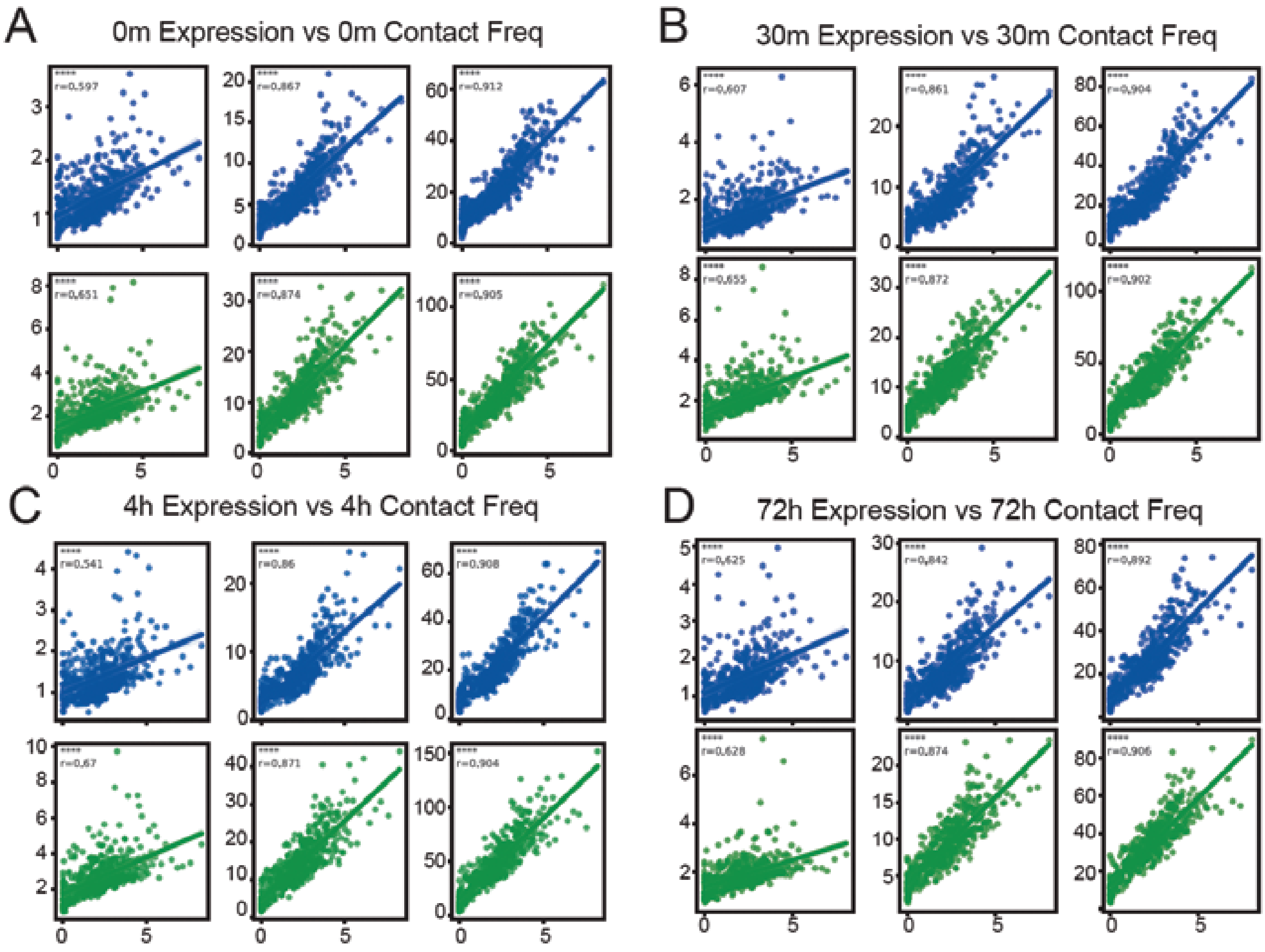

Supplementary Figure 9: **Binned (k=25) scatter plot of log expression (x-axis) vs. contact frequency (y-axis) according to the summarization function**. Columns are avg, max, sum respectively. The blue and green scatterplots represent H3K27ac and H3K4me3 **(A)** 0m **(B)** 30m **(C)** 4h **(D)** 72h data respectively. Correlation was calculated by linear regression. The significance was assessed by Spearman's rank correlation. For all data ns $p>0.05$, * $p<0.05$, ** $p<0.01$, *** $p<0.001$, **** $p<0.0001$.

Supp Fig 10

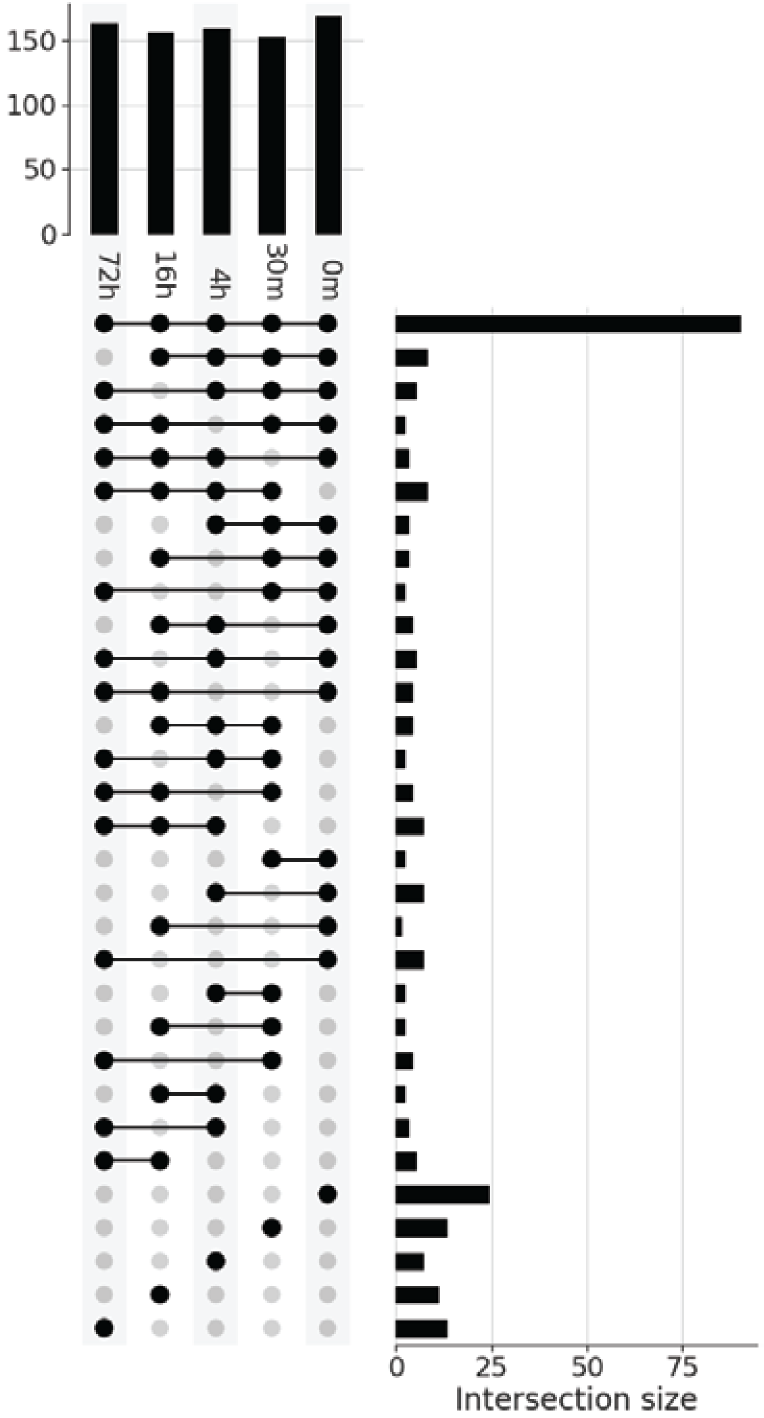

Supplementary Figure 10: **Number of dominant loops.** Dominant loops of AR-regulated genes are identified for every gene promoter by scaling contact frequency (H3K27ac or H3K4me3 separately) in range (0, 1), and selecting the highest ones with a threshold (0.8) at every time point (bar plot top). Most of the domintant loops are commonly found in every time point (upset plot).

Supp Fig 11

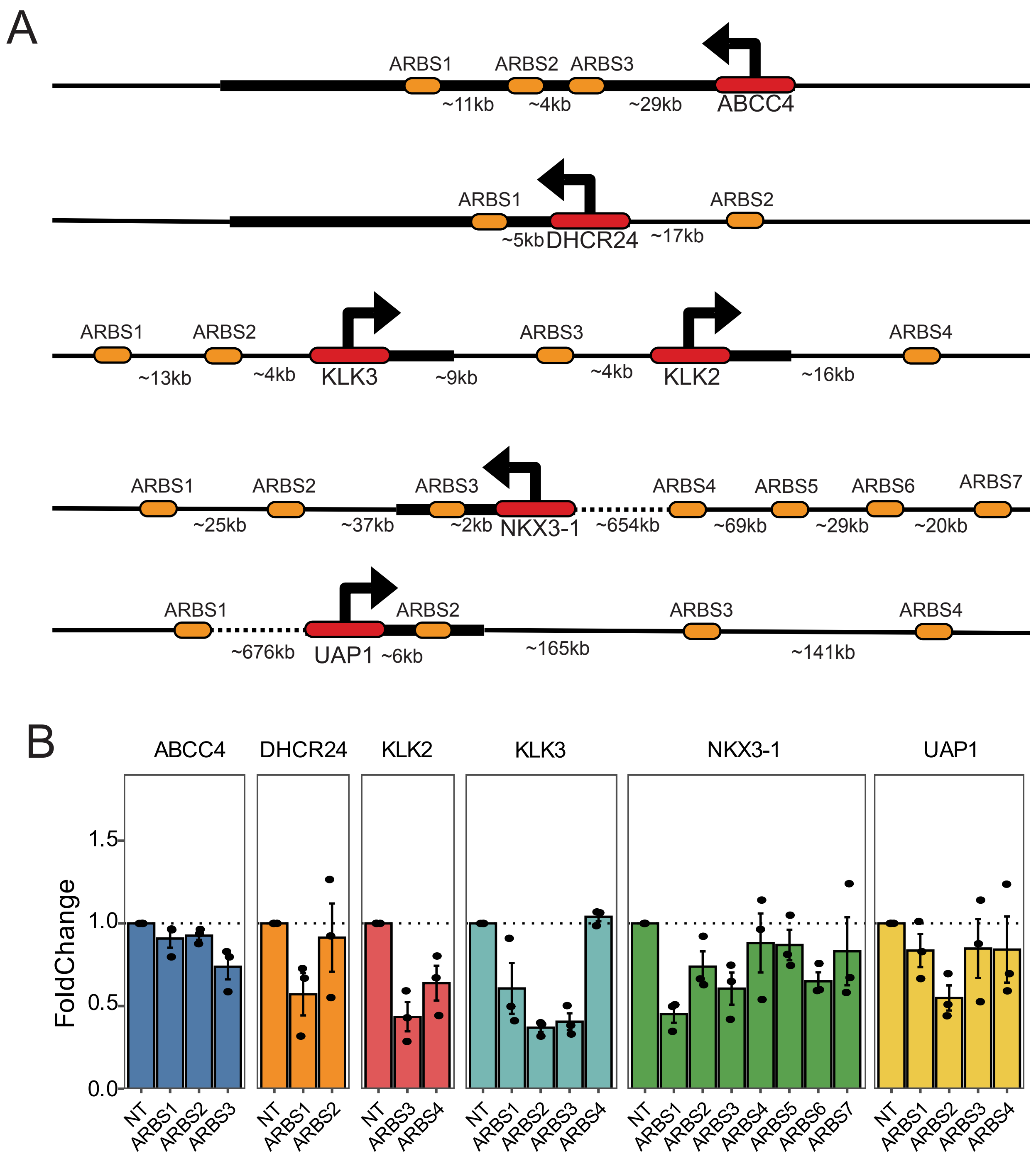

Supplementary Figure 11: **In total, 65 gRNA are desined for 20 ARBS (Supp Table 1). The experiment contains three biological replicates for each ARBS. (A)** Genomic locations of each enhancer tested with respect to gene promoters' location. **(B)** Gene expression changes in response to CRISPRi suppression of candidate enhancers.

Supp Fig 12

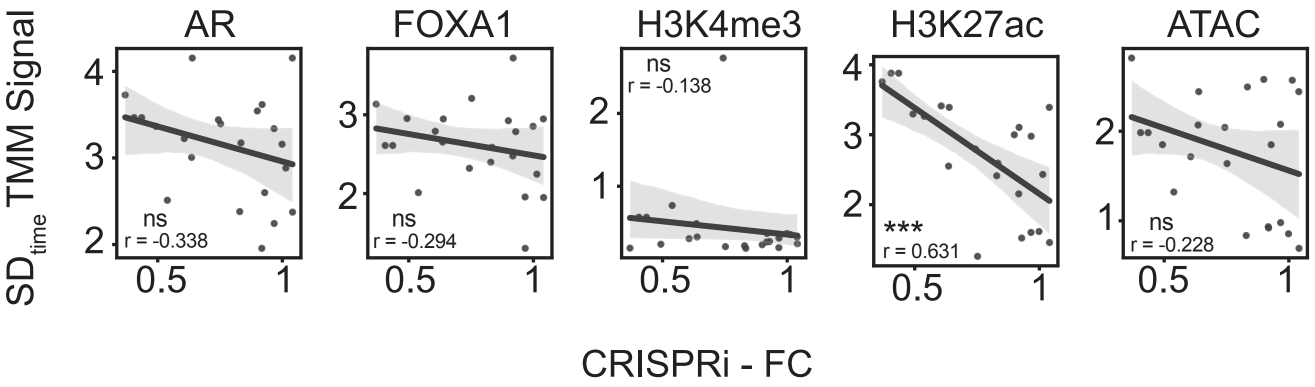

Supplementary Figure 12: **Changes in epigenetic features upon androgen treatment (SD$_{time}$) vs. Gene expression changes in response to CRISPRi suppression of candidate enhancers.** Correlation was calculated by linear regression. The significance was assessed by Spearman's rank correlation.

Supp Fig 13

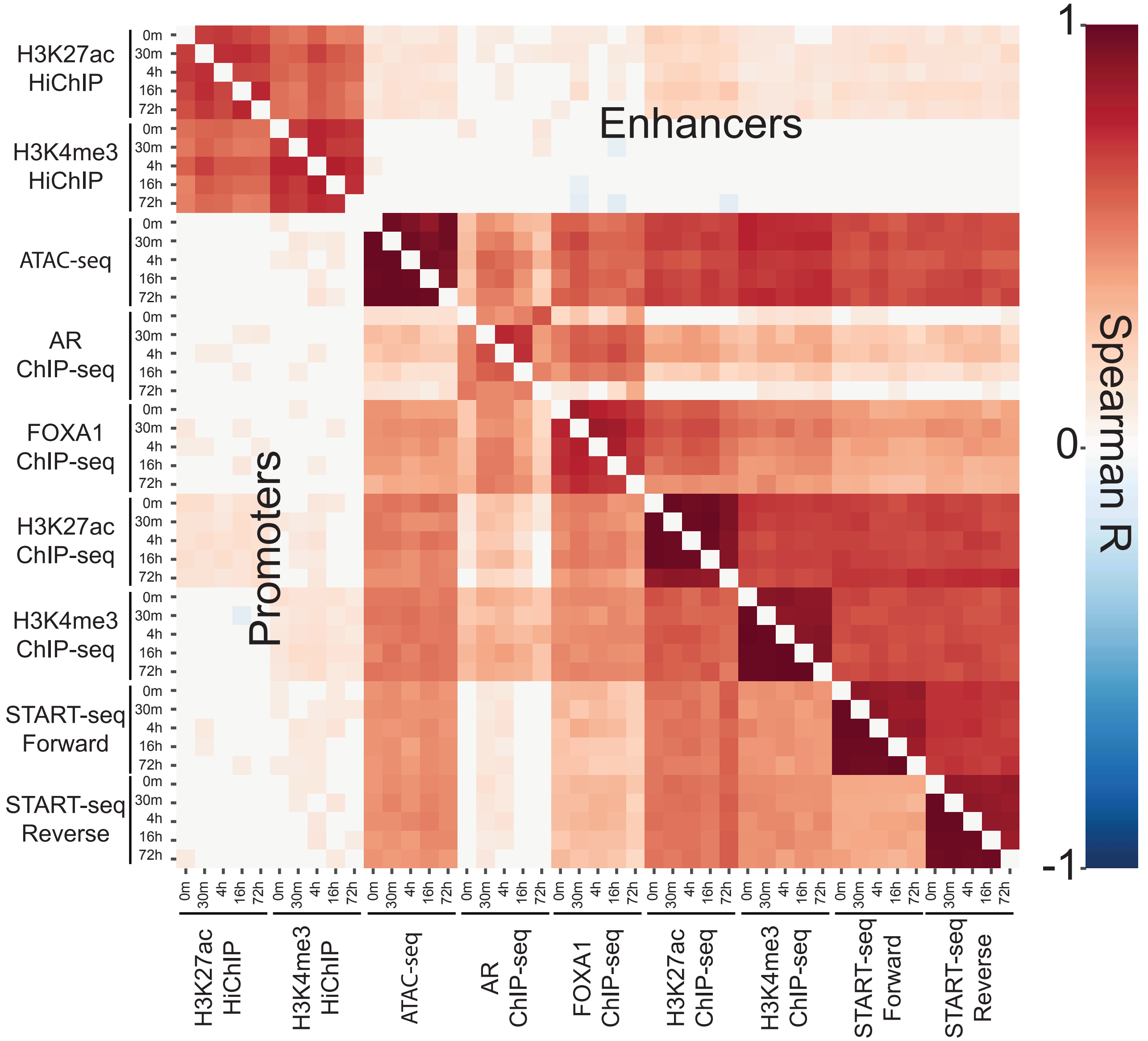

Supplementary Figure 13: Pairwise spearman correlation coefficient of features on promoters and their looped enhancers. Lower triangle represents the promoter features, and upper triangle represents the enhancer features.

Supp Fig 14

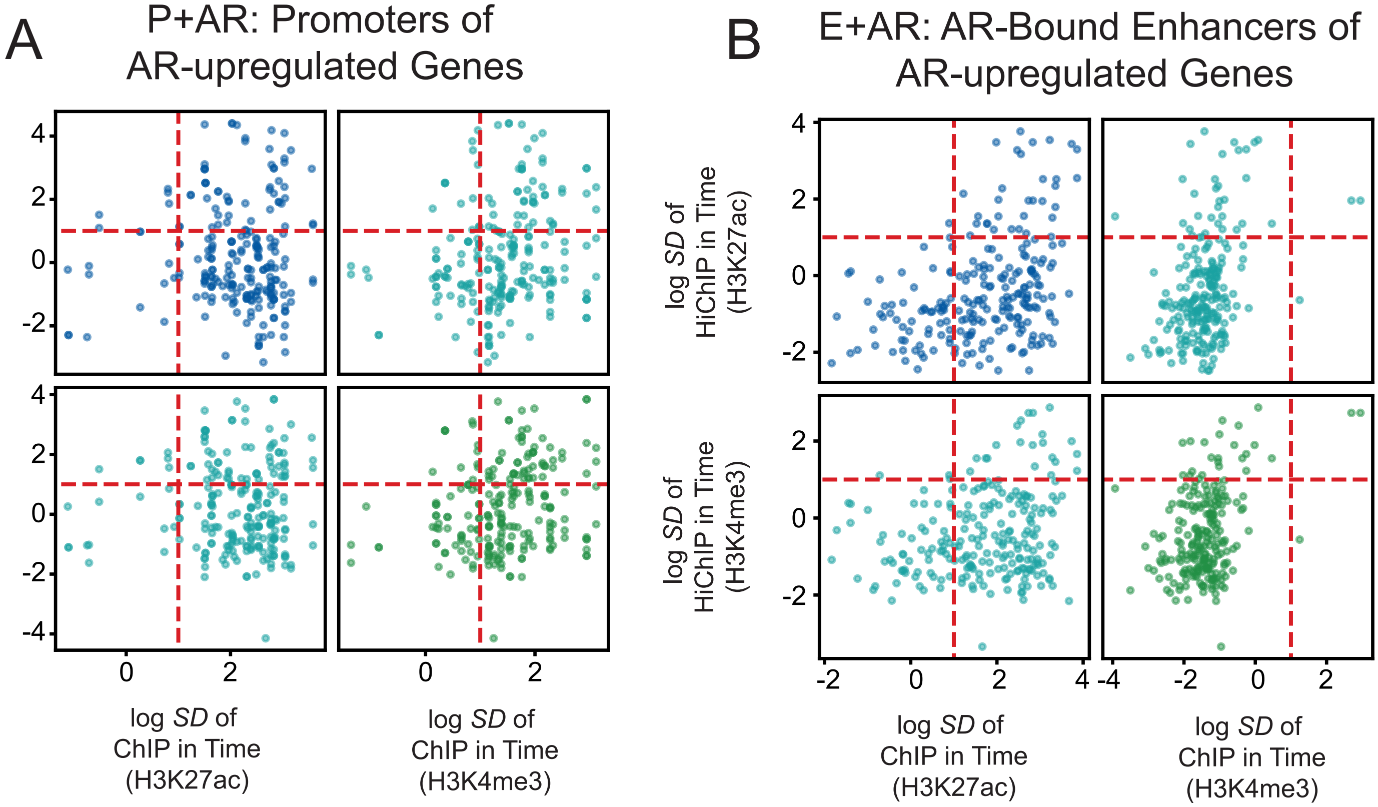

Supplementary Figure 14: Scatter plots representing kinetic changes in ChIP-seq (x-axis) and HiChIP (y-axis) of P-E pairs of which AR-upregulated genes and AR-bound enhancers. Top left panel H3K27ac HiChIP vs H3K27ac ChIP-seq. Top right panel H3K27ac HiChIP vs H3K4me3 ChIP-seq. Bottom left panel H3K4me3 HiChIP vs H3K27ac ChIP-seq. Bottom right panel H3K4me3 HiChIP vs H3K4me3 ChIP-seq. The red dashed lines represents the *log SD = 1*. **(A)** The ChIP-seq signal on promoters of AR-upregulated genes. **(B)** The ChIP-seq signal on AR-bound enhancers of AR-upregulated genes.
