# Supplementary material for: Decoding the epigenetics and chromatin loop dynamics of androgen receptor-mediated transcription
Source: Nat Commun. 2024 Nov 3;15:9494. doi: 10.1038/s41467-024-53758-5 (PMC11532539; doi:10.1038/s41467-024-53758-5)
Supplement: Supplementary file 3 — Description of Additional Supplementary Files [file 41467_2024_53758_MOESM3_ESM.pdf]

Description of Additional Supplementary Files

File Name: Supplementary Data 1
Description: The sequences of gRNAs and qRT-PCR primers for CRISPRi.

File Name: Supplementary Data 2
Description: The coordinates of gRNAs and qRT-PCR primers for CRISPRi. All coordinates are in hg19.

File Name: Supplementary Data 3
Description: Neighbors of androgen response genes and their dominance status (0m, 30m, 4h, 16h, 72h). All coordinates are in hg19.

File Name: Supplementary Data 4
Description: Neighbors of all genes and their dominance status with prostate cancer variation status (PMID: 37945903). All coordinates are in hg19.
