# Supplementary material for: Decoding the epigenetics and chromatin loop dynamics of androgen receptor-mediated transcription
Source: Nat Commun. 2024 Nov 3;15:9494. doi: 10.1038/s41467-024-53758-5 (PMC11532539; doi:10.1038/s41467-024-53758-5)
Supplement: Supplementary file 8 — Reporting Summary [file 41467_2024_53758_MOESM8_ESM.pdf]

nature portfolio

Corresponding author(s): Nathan Lack

Last updated by author(s): 09/26/2024

Reporting Summary

Nature Portfolio wishes to improve the reproducibility of the work that we publish. This form provides structure for consistency and transparency in reporting. For further information on Nature Portfolio policies, see our Editorial Policies and the Editorial Policy Checklist.

Statistics

For all statistical analyses, confirm that the following items are present in the figure legend, table legend, main text, or Methods section.

n/a	Confirmed	
☐	☒	The exact sample size (n) for each experimental group/condition, given as a discrete number and unit of measurement
☐	☒	A statement on whether measurements were taken from distinct samples or whether the same sample was measured repeatedly
☐	☒	The statistical test(s) used AND whether they are one- or two-sided<br>*Only common tests should be described solely by name; describe more complex techniques in the Methods section.*
☐	☒	A description of all covariates tested
☐	☒	A description of any assumptions or corrections, such as tests of normality and adjustment for multiple comparisons
☐	☒	A full description of the statistical parameters including central tendency (e.g. means) or other basic estimates (e.g. regression coefficient) AND variation (e.g. standard deviation) or associated estimates of uncertainty (e.g. confidence intervals)
☐	☒	For null hypothesis testing, the test statistic (e.g. F, t, r) with confidence intervals, effect sizes, degrees of freedom and P value noted<br>*Give P values as exact values whenever suitable.*
☒	☐	For Bayesian analysis, information on the choice of priors and Markov chain Monte Carlo settings
☒	☐	For hierarchical and complex designs, identification of the appropriate level for tests and full reporting of outcomes
☐	☒	Estimates of effect sizes (e.g. Cohen's d, Pearson's r), indicating how they were calculated

Our web collection on statistics for biologists contains articles on many of the points above.

Software and code

Policy information about availability of computer code

Data collection	No software was used for data collection.
Data analysis	Programming Languages:<br>Python (v3.9.7)<br>R (v4.1.1)<br><br>Data Processing:<br>- RNAseq:<br>STAR (v2.7.9)<br>Salmon (v0.14.1)<br>VIPER (https://github.com/hanfeisun/viper-rnaseq)<br>- ChIPseq & ATACseq:<br>ChiLin (https://github.com/cfce/chilin)<br>FastQC (v0.10.1)<br>BWA (v0.7.10)<br>MACS2 (v2.1.0.20140616)<br>bedGraphToBigWig (https://hgdownload.cse.ucsc.edu/admin/exe/)<br>Trim Galore (v0.5.0)<br>- HiChIP:

HiC-Pro (v3.1.0)
FitHiChIP (v10.0)

Data Analyses:
Bedtools (v2.30.0) - command-line
numpy (v1.21.6) - python
pandas (v1.5.3) - python
scipy (v1.10.0) - python
matplotlib (v3.6.2) - python
seaborn (v0.13.1) - python
pybedtools (v0.8.2) - python
pyBigWig (v0.3.18) - python
NetworkX (v3.1) - python
Cooler (v0.9.3) - python
GenomicInteractions (v1.34) - R

Code Availibility:
The code to reproduce the results from this study's data is available through the project's source code repository [https://github.com/birkiy/cisregulatorynetworks.git] and [https://github.com/birkiy/bluegill.git].

For manuscripts utilizing custom algorithms or software that are central to the research but not yet described in published literature, software must be made available to editors and reviewers. We strongly encourage code deposition in a community repository (e.g. GitHub). See the Nature Portfolio guidelines for submitting code & software for further information.

Data

Policy information about availability of data

All manuscripts must include a data availability statement. This statement should provide the following information, where applicable:

- Accession codes, unique identifiers, or web links for publicly available datasets
- A description of any restrictions on data availability
- For clinical datasets or third party data, please ensure that the statement adheres to our policy

The generated datasets are deposited to Gene Expression Omnibus (GEO), and are publicly availiable through GSE251898 [https://www.ncbi.nlm.nih.gov/geo/query/acc.cgi?acc=GSE251898] accession (ATAC-seq: GSE251893, ChIP-seq: GSE251894, HiChIP: GSE251895, RNA-seq: GSE251896, Start-seq: GSE251897). Additional data are provided with this paper through zenodo [https://zenodo.org/doi/10.5281/zenodo.13770484]. Source data are provided.

Research involving human participants, their data, or biological material

Policy information about studies with human participants or human data. See also policy information about sex, gender (identity/presentation), and sexual orientation and race, ethnicity and racism.

Reporting on sex and gender	This study does not include human participants.
Reporting on race, ethnicity, or other socially relevant groupings	This study does not include human participants.
Population characteristics	This study does not include human participants.
Recruitment	This study does not include human participants.
Ethics oversight	This study does not include human participants.

Note that full information on the approval of the study protocol must also be provided in the manuscript.

Field-specific reporting

Please select the one below that is the best fit for your research. If you are not sure, read the appropriate sections before making your selection.

☒ Life sciences ☐ Behavioural & social sciences ☐ Ecological, evolutionary & environmental sciences

For a reference copy of the document with all sections, see nature.com/documents/nr-reporting-summary-flat.pdf

Life sciences study design

All studies must disclose on these points even when the disclosure is negative.

Sample size	Sample sizes vary from assay to assay in this study. For ATAC-seq, H3K27ac ChIp-seq, H3K27ac HiChIP, RNA-seq and START-seq 2 replicates were used for every 5 time points. For AR, FOXA1, H3K4me3 ChIP-seq and H3K4me3 HiChIP 1 replicate was used for every 5 time points. In

total 75 experiments were performed.

The following quality metrics were assessed for each sample: (i) percentage of uniquely mapped reads, (ii) PCR bottleneck coefficient to identify potential over-amplification by PCR, (iii) FRiP (fraction of non-mitochondrial reads in peak regions), (iv) peak number, (v) number of peaks with 10-fold and 20-fold enrichment over the background, (vi) fragment size, (vii) the percentage of the merged peaks with promoter, enhancer, intron, or intergenic, and (viii) peak overlap with DNaseI hypersensitivity sites. For datasets with replicates, the replicate consistency was checked by two metrics: 1. Pearson correlation of reads across the genome using UCSC software wigCorrelate after normalizing signal to reads per million and 2. percentage of overlapping peaks in the replicates.

CRISPRi experiments 3 biological replicates were used.

Data exclusions: No data were excluded in the study.

Replication: For the omics data, replication is not applicable to in silico experiments in this study as the computed results are exact.

For the CRISPRi experiments, the standard deviation of biological replicates were given (Supp. Fig. 9B).

Randomization: This study generated data from a well studied cell-line. Therefore, randomization procedure was not relevant.

Blinding: This study generated data from a well studied cell-line. Therefore, blinding procedure was not relevant.

Reporting for specific materials, systems and methods

We require information from authors about some types of materials, experimental systems and methods used in many studies. Here, indicate whether each material, system or method listed is relevant to your study. If you are not sure if a list item applies to your research, read the appropriate section before selecting a response.

Materials & experimental systems

n/a	Involved in the study	
☐	☒	Antibodies
☐	☒	Eukaryotic cell lines
☒	☐	Palaeontology and archaeology
☒	☐	Animals and other organisms
☒	☐	Clinical data
☒	☐	Dual use research of concern
☒	☐	Plants

Methods

n/a	Involved in the study	
☐	☒	ChIP-seq
☒	☐	Flow cytometry
☒	☐	MRI-based neuroimaging

Antibodies

Antibodies used: Antibodies used for both ChIP-seq and HiChIP: Anti-Androgen Receptor antibody (0.1 µg/µL, Abcam, ab133273), Anti-FOXA1 antibody (1 µg/µL, Abcam, ab23738), Anti-H3K27ac antibody (2.8 µg/µL, Diagenode, C15410196), Anti-H3K4me3 antibody (1.3 µg/µL, Diagenode, C15410003).

Validation: Each company validated the antibodies using various methods, including dot plot, ChIP qPCR, Western blot, immuno-fluorescence, immunocytochemistry, and immunohistochemistry. Supplier-provided assay results and validation reports are available on the vendor’s website.

Eukaryotic cell lines

Policy information about cell lines and Sex and Gender in Research

Cell line source(s): LNCaP cells (#CRL-1740, ATCC)

Authentication: LNCaP cells were authenticated by sequencing and comparing short tandem repeats to parental LNCaP cells in ATCC database.

Mycoplasma contamination: Cells are routinely tested for Mycoplasma contamination. All tests were negative.

Commonly misidentified lines (See ICLAC register): LNCaP is not listed as being commonly misidentified.

Plants

Seed stocks: No plant species were used.

Novel plant genotypes: No plant species were used.

Authentication: No plant species were used.

ChIP-seq

Data deposition

☒ Confirm that both raw and final processed data have been deposited in a public database such as GEO.

☒ Confirm that you have deposited or provided access to graph files (e.g. BED files) for the called peaks.

Data access links
May remain private before publication.

https://www.ncbi.nlm.nih.gov/geo/query/acc.cgi?acc=GSE251898

Files in database submission

GSM7989602 LNCaP_AR_0m_ChIPseq
GSM7989603 LNCaP_AR_30m_ChIPseq
GSM7989604 LNCaP_AR_4h_ChIPseq
GSM7989605 LNCaP_AR_16h_ChIPseq
GSM7989606 LNCaP_AR_72h_ChIPseq
GSM7989607 LNCaP_FOXA1_0m_ChIPseq
GSM7989608 LNCaP_FOXA1_30m_ChIPseq
GSM7989609 LNCaP_FOXA1_4h_ChIPseq
GSM7989610 LNCaP_FOXA1_16h_ChIPseq
GSM7989611 LNCaP_FOXA1_72h_ChIPseq
GSM7989612 LNCaP_H3K27ac_0m_rep1_ChIPseq
GSM7989613 LNCaP_H3K27ac_0m_rep2_ChIPseq
GSM7989614 LNCaP_H3K27ac_30m_rep1_ChIPseq
GSM7989615 LNCaP_H3K27ac_30m_rep2_ChIPseq
GSM7989616 LNCaP_H3K27ac_4h_rep1_ChIPseq
GSM7989617 LNCaP_H3K27ac_4h_rep2_ChIPseq
GSM7989618 LNCaP_H3K27ac_16h_rep1_ChIPseq
GSM7989619 LNCaP_H3K27ac_16h_rep2_ChIPseq
GSM7989620 LNCaP_H3K27ac_72h_rep1_ChIPseq
GSM7989621 LNCaP_H3K27ac_72h_rep2_ChIPseq
GSM7989622 LNCaP_H3K4me3_0m_ChIPseq
GSM7989623 LNCaP_H3K4me3_30m_ChIPseq
GSM7989624 LNCaP_H3K4me3_4h_ChIPseq
GSM7989625 LNCaP_H3K4me3_16h_ChIPseq
GSM7989626 LNCaP_H3K4me3_72h_ChIPseq
GSM7989627 LNCaP_INPUT_0m_ChIPseq
GSM7989628 LNCaP_INPUT_30m_ChIPseq
GSM7989629 LNCaP_INPUT_4h_ChIPseq
GSM7989630 LNCaP_INPUT_16h_ChIPseq
GSM7989631 LNCaP_INPUT_72h_ChIPseq

Genome browser session
(e.g. UCSC)

Not applicable

Methodology

Replicates: For H3K27ac, 2 replicates were used; for AR, FOXA1, H3K4me3 1 replicate was used.

Sequencing depth: Each sample has >20 M 150-paired end reads.

Antibodies: Antibodies used for both ChIP-seq and HiChIP: Anti-Androgen Receptor antibody (0.1 µg/µL, Abcam, ab133273), Anti-FOXA1 antibody (1 µg/µL, Abcam, ab23738), Anti-H3K27ac antibody (2.8 µg/µL, Diagenode, C15410196), Anti-H3K4me3 antibody (1.3 µg/µL, Diagenode, C15410003).

Peak calling parameters	ChiLin pipeline was utilized to call peaks (https://github.com/cfce/chilin).
Data quality	The quality metrics are described in the methods section of the manuscript.
Software	Software listed above, and described in the methods section of the manuscript.
